# Supplementary material for: Strong, tough, rapid-recovery, and fatigue-resistant hydrogels made of picot peptide fibres
Source: Nat Commun. 2023 May 4;14:2583. doi: 10.1038/s41467-023-38280-4 (PMC10160100; doi:10.1038/s41467-023-38280-4)
Supplement: Supplementary file 1 — Supplementary Information [file 41467_2023_38280_MOESM1_ESM.pdf]

Supplementary Information for

Strong, tough, rapid-recovery, and fatigue-resistant hydrogels made of picot peptide fibres

Bin Xue[†], Zoobia Bashir [†], Yachong Guo, Wenting Yu, Wenxu Sun, Yiran Li, Yiyang Zhang, Meng Qin, Wei Wang[*] and Yi Cao[*]

***Correspondence to: caoyi@nju.edu.cn or wangwei@nju.edu.cn**

This PDF file includes:

Supplementary Methods
Supplementary Figures 1 to 23
Supplementary Tables 1 to 2

Table of contents

Supplementary Methods ... 3
- **Prediction of the GK_{11} structure and GK_{11}-Cu^{2+} binding** ... 3
- **Circular dichroism (CD) spectroscopy measurements** ... 3
- **Fourier transform infrared (FT-IR) spectroscopy measurements** ... 4
- **Ultraviolet visible (UV–vis) spectroscopy measurements** ... 4
- **Estimation of peptide concentrations in hydrogels** ... 4
- **X-ray fluorescence spectroscopy (XRFS) measurements** ... 5
- **AFM based single molecule force spectroscopy (SMFS) measurements** ... 5
- **Field emission scanning electron microscope (FESEM)** ... 6
- **Swelling measurements** ... 6
- **Water content of the hydrogels** ... 6
- **Measurement of toughness** ... 7
- **Using EDTA and GuHCl to perturbing the metal ion-clad picot fibres** ... 7
- **Cu^{2+} release from hydrogels** ... 7
- **Calculation of the hidden length in the hydrogel network** ... 8

Supplementary Figures ... 11
- **Supplementary Figure 1** ... 11
- **Supplementary Figure 2** ... 12
- **Supplementary Figure 3** ... 12
- **Supplementary Figure 4** ... 13
- **Supplementary Figure 5** ... 14
- **Supplementary Figure 6** ... 15
- **Supplementary Figure 7** ... 16
- **Supplementary Figure 8** ... 16
- **Supplementary Figure 9** ... 17
- **Supplementary Figure 10** ... 17
- **Supplementary Figure 11** ... 17
- **Supplementary Figure 12** ... 18
- **Supplementary Figure 13** ... 18
- **Supplementary Figure 14** ... 19
- **Supplementary Figure 15** ... 20
- **Supplementary Figure 16** ... 21
- **Supplementary Figure 17** ... 22
- **Supplementary Figure 18** ... 23
- **Supplementary Figure 19** ... 24
- **Supplementary Figure 20** ... 25
- **Supplementary Figure 21** ... 26
- **Supplementary Figure 22** ... 27
- **Supplementary Figure 23** ... 28

Supplementary Tables ... 29
- **Supplementary Table 1** ... 29
- **Supplementary Table 2** ... 30

Supplementary References ... 30

Supplementary Methods

Prediction of the GK_{11} structure and GK_{11}-Cu^{2+} binding

The structure prediction of GK_{11} was obtained by employing Alphafold2[1] neural network through ColabFold pipline [2]. Different number GK_{11} ranging from 2 to 40 was predicted with default setting. Random coiled ploy-glycine were used to linked neighbouring GK_{11} from head to end. The sequence is aligned though MMseqs2 search tool[3]. The structural relaxation of final structure is equipped with AMBER14 force fields[4]. The simulations were carried out at constant temperature (300 K) and constant pressure (1 bar) in the Gromacs package[5]. Water molecules were modelled using the TIP3P model. The SETTLE algorithm[6] was applied to constrain bond lengths and angles of water molecules, and LINCS algorithm[7] was used to constrain all other bond lengths, allowing a time step of 2 fs. Each simulation was optimized, equilibrated and continuously run in the NPT ensemble. The binding position of Cu^{2+} is predicted with MIB server[8] and again optimized with Gromacs. The GK_{11}-Cu^{2+} binding structure was used to calculate the binding free energy with MM-PBSA method[9]. For all trajectory with Amber15[10], the decomposition of binding free energy was calculated by taking into account of the enthalpic part containing: Electrostatic, Van der Waals, Polar and Non-Polar Solvation.

$$\Delta G_{bind} = \Delta E_{ele} + \Delta G_{vdW} + \Delta G_{pol} + \Delta G_{nonpol} \quad (1)$$

Circular dichroism (CD) spectroscopy measurements

The CD spectra was recorded to investigate the secondary structure of the self-assembled peptide in the absence and presence of Cu^{2+}. Typically, a stock solution of $Cu(NO_3)_2$ at the concentration of 15 mM was prepared using deionized water. Then, GK_{11} was dissolved into the $Cu(NO_3)_2$ solution to the concentration of 15 mM. The resulting solutions were stored at room temperature (22 °C) for 2 hours, and diluted for 40 times. The CD spectra of all samples were recorded using a J-815 (JASCO Inc., Japan) spectrophotometer. The cuvette width was 1 mm, and the bandwidth was 0.2 nm.

Fourier transform infrared (FT-IR) spectroscopy measurements

The IR spectra of the peptide solutions (45 mM) or wet hydrogel samples in the presence and absence of Cu^{2+} were recorded using a NICOLET iS10 (NICOLET, USA) spectrometer directly without drying. The reported spectra were the average of more than 60 scans to increase the signal-to-noise ratio with background signals subtracted.

Ultraviolet visible (UV–vis) spectroscopy measurements

For the calibration curve of peptide concentration, the GK_{11} peptide was dissolved in 1 M Tris buffer (pH=7.60) to different concentrations (0.08-0.24 mg mL^{-1}) and UV–vis spectra of the solutions were recorded using a V-550 (JASCO Inc., Japan) spectrophotometer. The cuvette width was 1 mm, and the bandwidth was 0.2 nm.

For the determination of the binding stoichiometry of Cu^{2+} to GK_{11}, the peptide was dissolved in 1 M Tris buffer (pH=7.60, containing 300 mM KCl) containing different concentrations of $CuCl_2$ (0, 0.1, 0.2, 0.3, 0.4, 0.5, 0.6 and 0.7 mM) to the concentration of 0.5 mM. The UV–vis spectra of the solutions were recorded using a V-550 (JASCO Inc., Japan) spectrophotometer. The peak fitting of the spectra was accomplished with Peakfit (v4.15.0).

Estimation of peptide concentrations in hydrogels

The initial peptides mass added to the reaction mixtures to prepare the hydrogels were recorded as W_1. After the UV illumination during the preparation of the hydrogel, the pregels were immersed in a 10 × gel volume of 1 M Tris buffer (pH=7.60, containing 300 mM KCl) and allowed to equilibrate for more than 24 hours. Then, the mass of the peptides (W_2) not incorporated in the hydrogel networks was estimated based on the UV–vis absorbance at 212 nm of the leachate and the peptide calibration curves based on UV–vis spectra (Supplementary Fig. 5). The incorporated peptides were calculated as $\sigma=(W_1-W_2)/W_1◊100\%$. The integration efficiency of the GK_{11} peptide in the hydrogels was higher than 98%.

X-ray fluorescence spectroscopy (XRFS) measurements

The mass ratio of copper in the GK_{11}-Cu^{2+} binding was measured using a Thermo ARL9800XP + X-ray fluorescence (XRF) spectrometer. The p-Pep/Cu^{2+} hydrogels at different concentrations of GK_{11} were prepared. After dialyzed in 1 M Tris buffer (pH=7.60, containing 300 mM KCl) to remove the unbinding Cu^{2+}, the hydrogels were lyophilized. Then, the dry samples were finely ground and pressed into slices prior to the measurements, and determined directly using a semiquantitative method based on XRFS.

AFM based single molecule force spectroscopy (SMFS) measurements

For the preparation of cantilevers used in SMFS, silicon nitride (Si_3N_4) cantilevers (MLCT, Bruker, USA) were first cleaned with Milli-Q water, and then placed in a chromic mixture (chromic acid) at 80 °C for 30 min. After that, the cantilevers were washed with deionized water, then ethanol, and dried under a steam of nitrogen. For the preparation of metal ion-clad picot fibres absorbed on glass substrates used in SMFS, ACLT-GK_{11} (43.2 mM) and acrylamide (703 mM or 1406 mM) were copolymerized in deionized water and diluted for 3 times using 1 M Tris buffer (pH=7.60, containing 300 mM KCl and 150 mM $CuCl_2$) to induce self-assembly and ion binding. For the preparation of substrates, the glass substrates were cut into 1 × 1 cm^2 slides and soaked in a freshly prepared chromic mixture overnight. Then the substrates were washed with deionized water and ethanol, and then dried under a steam of nitrogen. The as prepared ion-clad picot fibre solution (50 μL) was dropped on the glass substrate and allowed to absorb for 30 min. The solution was removed, and the substrate was washed using Tris buffer for 3 times to remove the unabsorbed fibres.

AFM force spectroscopy experiments were carried out on a commercial AFM (JPK Nanowizard II). The force–distance curves were recorded by commercial software from JPK and analyzed by custom-written procedures in Igor pro 6.37 (Wavemetrics, Inc.). All the experiments were conducted at the room temperature (22 °C) and performed in

1 M Tris buffer (pH=7.60, containing 300 mM KCl). Soft silicon nitride MLCT-D cantilevers with typical spring constants of 30–45 pN nm^{-1} were used for all experiments and calibrated using the thermal tune method. Typically, the cantilever was brought in contact with the substrate and held at the surface for 3 s, then retracted at a constant velocity of 2.0 μm s^{-1}. The sampling rate was 8 kHz. The contour length increment was determined by fitting the force peaks using the worm-like chain model with persistence lengths in a range of ~0.2–0.4 nm. The contour length increment of each peak (ΔL) was considered as the hidden length between peptides.

Field emission scanning electron microscope (FESEM)

The FESEM images were obtained using a Quanta scanning electron microscope (GeminiSEM360, Zeiss, Germany) at 15 kV. The hydrogels were dialyzed in Milli-Q water for 24 h to remove the unbound salts and lyophilized prior to the measurement.

Swelling measurements

In a typical swelling experiment, the volume of the pregels after UV illumination was denoted the initial volume (V_1), and the volume of hydrogel after dialysis in 1 M tris buffer (pH=7.60, containing 300 mM KCl) was denoted the swollen volume (V_2). The swelling ratio was calculated as $V_2/V_1 \times 100\%$.

Water content of the hydrogels

The hydrogels were weighed with the wet weight recorded as W_1. Then, the hydrogel samples were dialyzed in Milli-Q water for 24 h to remove the unbound salts and lyophilized. The dry weight was recorded again as W_2. The water content was calculated as $(W_1-W_2)/W_1 \times 100\%$.

Measurement of toughness

The toughness of hydrogel was determined as fracture energy and measured with pure shear tests (Supplementary Fig. 13) [11-14]. Typically, two different samples of the same size, notched and unnotched, were stretched until cracking. The width of the samples was 8 mm. The crack length of the notched sample was 1.6 mm. During stretching, the initial distance between the two clamps was set as 3 mm (L_0), and the distance between the two clamps when the crack started to propagate in the notched sample was recorded as L_c. The fracture energy was calculated from $W = U(L_c) \times L_0$, where $U(L_c)$ is the area below the tensile stress-strain curve of the unnotched sample at the critical stretching distance L_c. The onset of crack propagation of the notched sample was determined using a camera.

Using EDTA and GuHCl to perturbing the metal ion-clad picot fibres

To perturb the metal chelation in picot fibres using EDTA, the p-Pep/Cu^{2+} hydrogels were immersed in EDTA solutions (100 mM in 1 M Tris buffer containing 300 mM KCl, pH=7.60) for 24 h, and the Cu^{2+} ion in the hydrogels was removed by the competitive binding of EDTA. To recharge the hydrogel with Cu^{2+} ions, the hydrogels were dialyzed in 1 M Tris buffer (pH=7.60, containing 300 mM KCl and 200 mM $CuCl_2$) for more than 24 h to reform the coordination bonds.

To perturb the picot metal ion-clad fibres using GuHCl, the p-Pep/Cu^{2+} hydrogels were immersed into GuHCl solutions (1.2 M in 1 M Tris buffer containing 300 mM KCl, pH=7.60) for 24 h, and the self-assembled of peptide in the hydrogels was destroyed by GuHCl. To reconstruct the peptide assembly, the hydrogels were dialyzed in 1 M Tris buffer (pH=7.60, containing 300 mM KCl and 200 mM $CuCl_2$) for more than 24 h.

Cu^{2+} release from hydrogels

For the detecting of Cu^{2+} release from p-Pep/Cu^{2+} hydrogels, a hydrogel was subjected to stretching-relaxation cycles. After different cycles, the hydrogel was immersed into

1 M Tris buffer (pH=7.60, containing 300 mM KCl and 0.3 mM sodium diethyldithiocarbamatre (DDTC-Na)) immediately for 30 min. The volume of Tris buffer was five times of the p-Pep hydrogel. Then the UV–vis spectra of the leachates were recorded using a V-550 (JASCO Inc., Japan) spectrophotometer. The absorbance at 452 nm was used to monitor the concentration of Cu^{2+}.

Calculation of the hidden length in the hydrogel network

Considering the network of the p-Pep/Cu^{2+} hydrogel, the average length between two neighbouring cross-linking points before the release of the hidden length (top of Supplementary Fig. 18) can be determined as Eq. (2).

$$L_1 = L_{\text{acrylamide+pep}} + 2R_{PEG} \qquad (2)$$

where $L_{\text{acrylamide+pep}}$ is the length of the picot fibre between two neighbouring cross-linking points and R_{PEG} is the mean square end-to-end distance of a linear PEG (5 kDa). $L_{\text{acrylamide+pep}}$ can be determined as $\frac{C_{\text{pep}} \times (W_{pep} + L_{gap})}{4C_{PEG}}$, in which C_{pep} is the molar concentration of ACLT-GK_{11} peptide, W_{pep} is the average width of an ACLT-GK_{11} peptide (width of a β sheet), L_{gap} is the average length of the gap between adjacent peptide in the assembled GK_{11}, and C_{PEG} is the molar concentration of 4-armed PEG-ACLT. Following the worm-like chain (WLC) model, R_{PEG} is determined as $R_{PEG} = \sqrt{2\zeta_{PEG} L_{PEG}}$, in which ζ_{PEG} and L_{PEG} are the persistence length and the contour length of PEG, respectively. We used the ζ_{PEG} of 0.38 nm and L_{PEG} of 40 nm according to the previous literatures.[15-17] The average width of a peptide (width of the β sheet) was set as 0.35 nm and the gap between adjacent peptide was 0.30 nm according to the simulation.

The average length between two neighbouring cross-linking points after the release of the hidden length (bottom of Supplementary Fig. 18) can be determined as Eq. (3).

$$L_2 = R_{\text{acrylamide+pep}} + 2R_{PEG} \qquad (3)$$

in which $R_{\text{acrylamide+pep}}$ is the mean square end-to-end distance of copolymerized acrylamide and peptide, R_{PEG} is the mean square end-to-end distance of PEG. The mean square end-to-end distance of copolymerized acrylamide and peptide can be determined following the WLC model.

$$R_{\text{acrylamide+pep}} = \sqrt{2\zeta_{\text{acrylamide+pep}}\, L_{\text{acrylamide+pep}}} \qquad (4)$$

where $\zeta_{\text{acrylamide+pep}}$ and $L_{\text{acrylamide+pep}}$ are the persistence length and contour length of copolymerized acrylamide and peptide, respectively. 0.4 nm was used as the persistence length of copolymerized acrylamide and peptide.[18] The contour length of copolymerized acrylamide and peptide can be determined as Eq. (5).

$$L_{\text{acrylamide+pep}} = \frac{C_{\text{acrylamide}}+C_{pep}}{4C_{PEG}} \times 2L_{C-C} \qquad (5)$$

where L_{C-C} is the length of the C-C bond, $C_{\text{acrylamide}}$, C_{pep} and C_{PEG} are the molar concentrations of acrylamide, ACLT-GK_{11} and PEG. The length of C-C bond was considered as 0.15 nm. Finally, the theoretical ratio of the average length between two neighbouring cross-linking points after and before the release of the hidden length was calculated as $\frac{L_2}{L_1}$ =2.0.

Based on the classical rubber elasticity theory and Gaussian chain statistics[19-22], the stress of the hydrogel can be described as Eq. (6).

$$\sigma = G\left(\lambda - \frac{1}{\lambda^2}\right) = \frac{\rho RT}{M_c}\left(\lambda - \frac{1}{\lambda^2}\right) \qquad (6)$$

in which σ is the stress, $G = \frac{\rho RT}{M_c}$ is the shear modulus, ρ is the density of the polymer in the network, M_c is the molecular weight between two neighbouring cross-linking points, R is the gas constant, and λ is the extension ratio. Young's modulus E is defined as $E = \frac{\partial\sigma}{\partial\lambda}$ and $E=3G$ when $\lambda=1$. The ratio of Young's modulus for the same hydrogel before and after the release of the hidden length is $\varepsilon = \frac{\rho_1 M_{c2}}{\rho_2 M_{c1}}$. According to the Young's modulus and swelling ratio of the hydrogel before and after the treatment of GuHCl in experiments, the ratio of M_c after and before the release of the hidden length is ~ 1.8

± 0.3. We assume that the length of the polymer chain in the hydrogel network is directly proportional to its molecular weight. As a result, the ratio of the average length between two neighbouring cross-linking points after and before the release of the hidden length in experiments was calculated as ~ 1.8 ± 0.3.

Supplementary Figures

a

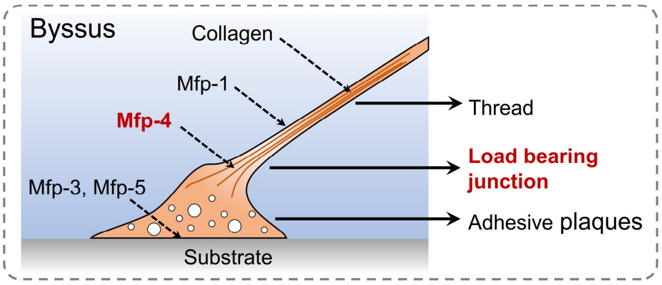

b

HVHGHHLLHR	HVHRHSHLHG	HVHMHRVSHR	IMHRHRVLHG	70
HVHRHRVLHN	HVHRHSVLHG	HVHRHRVLHR	HVHRHNVLHG	110
HVHRHRVLHK	HVHNHRVLHK	HLHKHQVLHG	HVHRHQVLHK	150
HVHNHRVLHK	HLHKHQVLHG	HVHTHRVLHK	HVHKHRVLHK	190
HLHKHQVLHG	HIHTHRVLHK	HLHKHQVLHG	HVHTHRVLHK	230
HVHKHRVLHK	HLHKHQVLHG	HVHMHRVLHK	HVHKHRVLHK	270
HVHKHHVVHK	HVHSHRVLHK	HVHKHRVEHQ	HVHKHHVLHR	310
HVHSHHVVHS	HVHKHRVVHS	HVHKHNVVHS	HVHRHQILHR	350
HVHRHQVVHR	HVHRHLIAHR	HIHSHQAAVH	RHVHTHVF	388

c

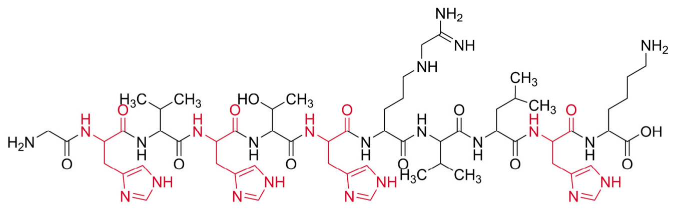

d

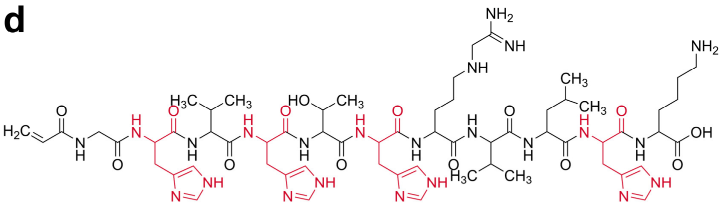

Supplementary Figure 1. Schematic of the mussel byssus structures (a), histidine rich peptide sequence of Mfp-4 (b), and chemical structures of GK_{11} (c) and ACLT-GK_{11} (d) peptides. Histidine in the peptide sequence is highlighted in red.

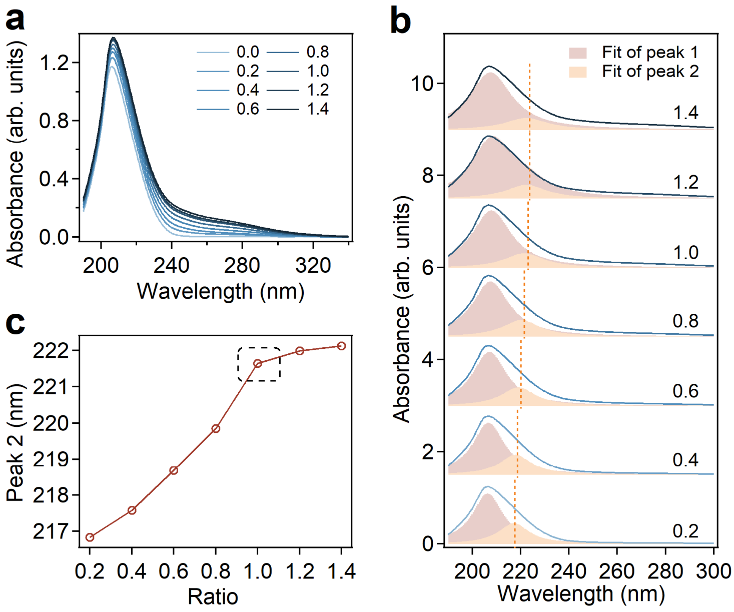

Supplementary Figure 2. Ultraviolet visible (UV–vis) spectroscopy of the GK_{11} peptide with Cu^{2+}. a, UV–vis spectra of mixtures with different levels of Cu^{2+}: GK_{11} ratios (molar ratios of 0.0, 0.2, 0.4, 0.6, 0.8, 1.0, 1.2 and 1.4). The concentration of GK_{11} was 0.6 mg mL^{-1}. b, Fits for the double peaks of UV–vis spectra for GK_{11} with different levels of Cu^{2+}: GK_{11} ratios using a bimodal distribution based on a Gaussian function. c, Shifts in peak 2 determined by fitting the UV–vis spectra with a bimodal distribution based on a Gaussian function. The slope of the shift curve varied sharply at a ratio of 1.0, indicating the binding stoichiometry of Cu^{2+} to GK_{11}.

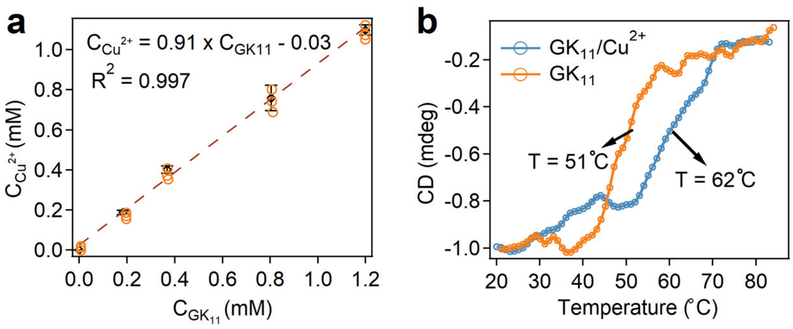

Supplementary Figure 3. XRFS and varied temperature CD spectrum of GK_{11} peptide without and with Cu^{2+} ions. a, Molar ratios of Cu^{2+} ions and the GK_{11} peptide at different GK_{11} concentrations determined by XRFS. The black dots were mean values

calculated from XRFS data (n=3), and the yellow dashed line was a linear fit. Error bars represent the standard deviation (n = 3 independent samples). b, Normalized varied temperature CD spectrum of the GK_{11} peptide without and with Cu^{2+} ions. The monitoring wavelength was 220 nm.

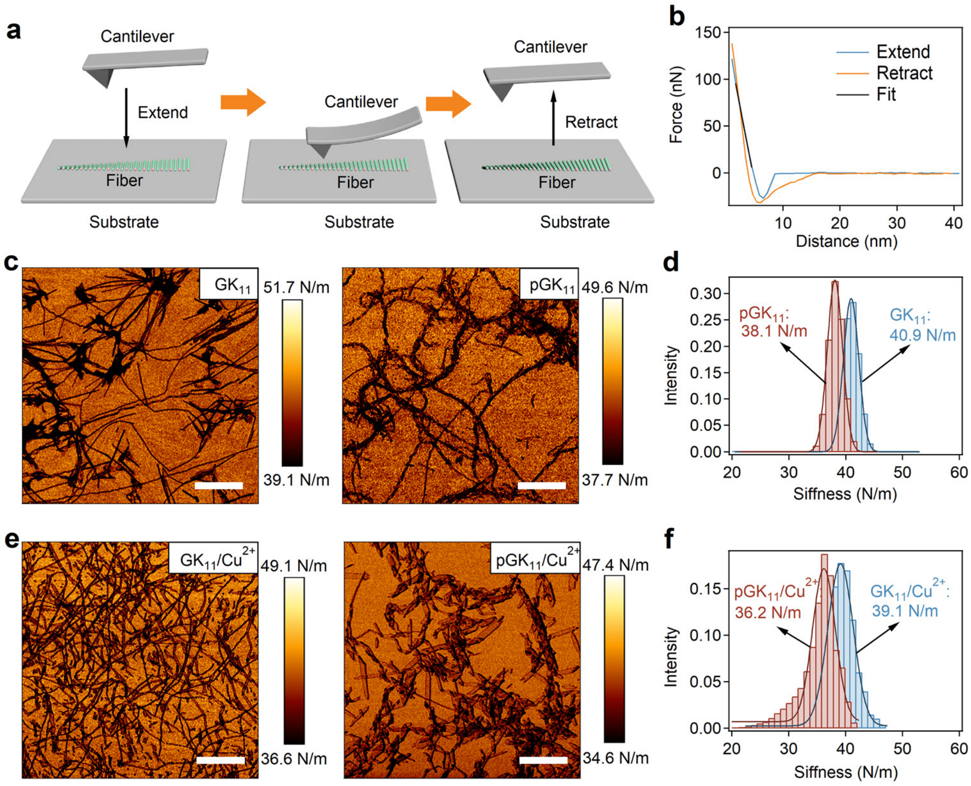

Supplementary Figure 4. Mechanical strengths of the peptide fibres and picot fibres. a, Schematic of the AFM-based nanoindentation test for nanofibres. b, Typical force-displacement curves from the AFM-based nanoindentation experiments. The stiffness was determined by fitting the extend-displacement curve. c, d, Topographic stiffness maps (c) and statistical stiffness distributions (d) of GK_{11} and pGK_{11} fibres. Scale bar =1 μm. e, f, Topographic stiffness maps (e) and statistical stiffness distributions (f) of GK_{11}/Cu^{2+} and pGK_{11}/Cu^{2+} fibres. Scale bar =1 μm.

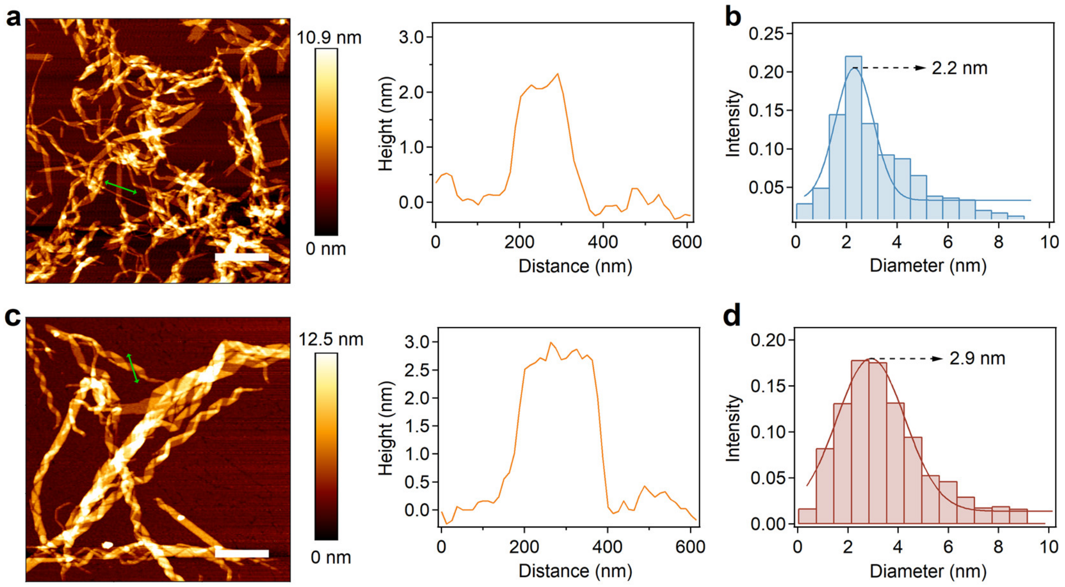

Supplementary Figure 5. AFM imaging of metal ion-clad picot fibres (pGK$_{11}$/Cu^{2+}) at different acrylamide:ACLT-GK$_{11}$ ratios. a, AFM image (left) and height profile (right) of metal ion-clad picot fibres (pGK$_{11}$/Cu^{2+}) at the acrylamide:ACLT-GK$_{11}$ ratio of 16.3. The height profile at right corresponds to the cross line (green) in the left image. Scale bar =1 μm. b, Diameter distribution of metal ion-clad picot fibres (pGK$_{11}$/Cu^{2+}) at the acrylamide:ACLT-GK$_{11}$ ratio of 16.3. c, AFM image (left) and height profile (right) of metal ion-clad picot fibres (pGK$_{11}$/Cu^{2+}) at the acrylamide:ACLT-GK$_{11}$ ratio of 32.6. The height profile at right corresponds to the cross line (green) in the left image. Scale bar =1 μm. d, Diameter distribution of metal ion-clad picot fibres (pGK$_{11}$/Cu^{2+}) at the acrylamide:ACLT-GK$_{11}$ ratio of 32.6.

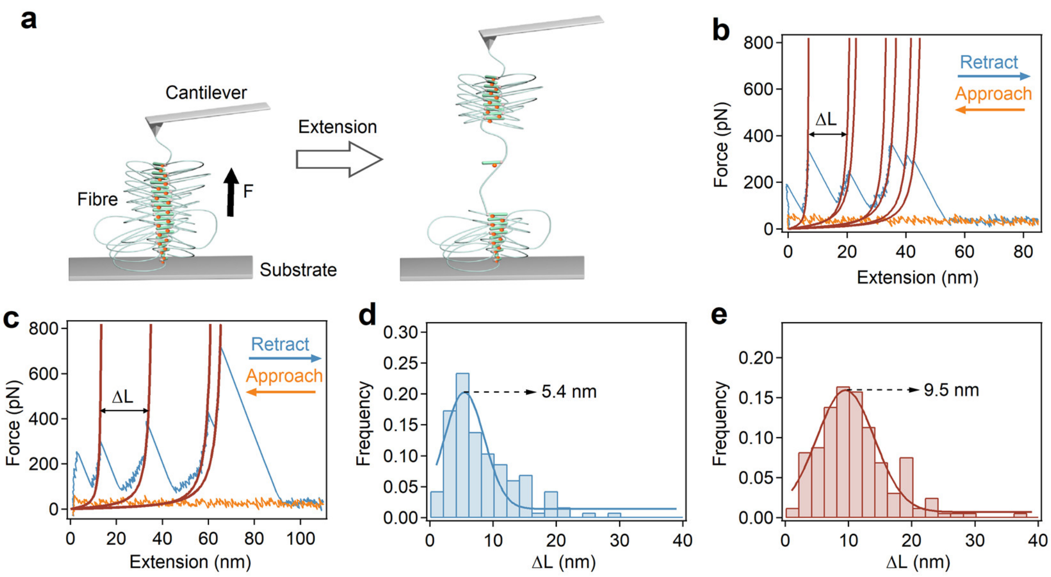

Supplementary Figure 6. SMFS of metal ion-clad picot fibres at different ratios of acrylamide and ACLT-GK$_{11}$. a, Schematic of the AFM-based SMFS for metal ion-clad picot fibres. b, c, Representative force–distance curves for the rupture of the metal ion-clad picot fibre at acrylamide:ACLT-GK$_{11}$ ratios of 16.3 (b) and 32.6 (c). The pulling speed was 2 μm s^{-1} and each peak corresponds to an individual rupture event between a pair of peptide strands. Red lines correspond to worm-like chain (WLC) fitting to the rupture events using the persistence length of 0.2-0.4 nm. ΔL indicates the hidden length between fractured peptides. d, e, Histograms of hidden lengths in metal ion-clad picot fibres at the acrylamide:ACLT-GK$_{11}$ ratio of 16.3 (d) and 32.6 (e). The measured hidden lengths (ΔL) were 5.4 nm (N=230) and 9.5 nm (N=316), respectively. Red lines correspond to a Gaussian fit.

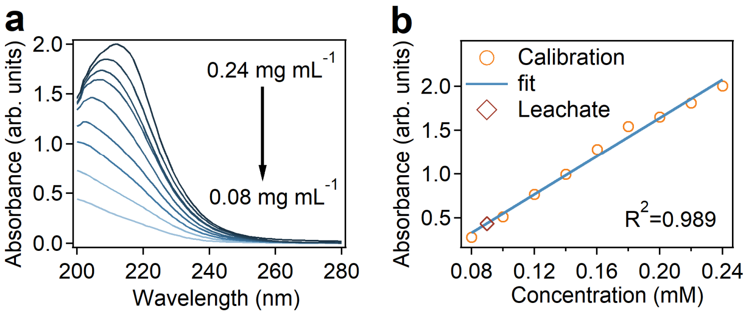

Supplementary Figure 7. Estimation of the integration efficiency of the GK_{11} peptide in the hydrogels. a, UV–vis spectra of the GK_{11} peptide at different concentrations in water. b, Calibration curve of GK_{11} peptide concentrations and OD_{212nm} (UV-vis absorbance at 212 nm). The blue line corresponds to a linear fit, the yellow points correspond to the OD_{212nm} values, and the red points correspond to the peptide concentration of the hydrogel leachate. The integration efficiency of the peptide in hydrogels was greater than 98%.

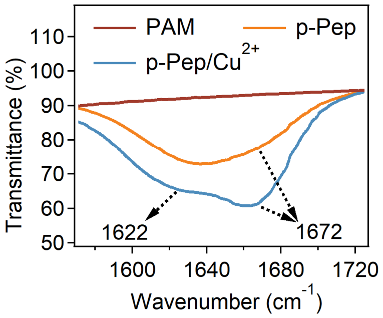

Supplementary Figure 8. Fourier transform infrared (FT-IR) spectra of PAM, p-Pep and p-Pep/Cu^{2+} hydrogels. Peaks similar to those observed in the GK_{11} and GK_{11}/Cu^{2+} solutions suggested the occurrence of peptide self-assembly inside the hydrogels.

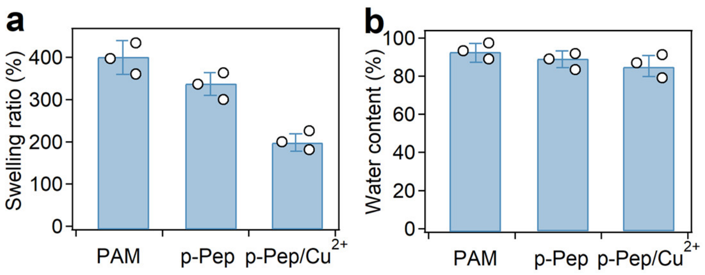

Supplementary Figure 9. Swelling ratios (a) and water contents (b) of PAM, p-Pep and p-Pep/Cu^{2+} hydrogels. Values represent the mean and standard deviation (n = 3 independent samples).

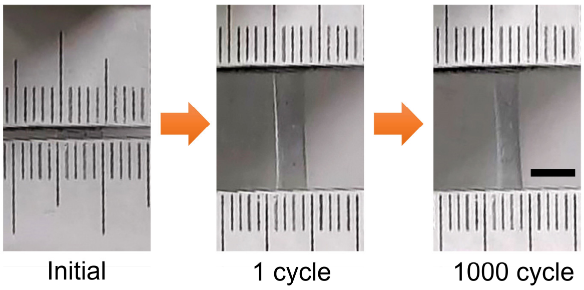

Supplementary Figure 10. Images of initial and stretched p-Pep/Cu^{2+} hydrogels after different numbers of stretching cycles (strain ~15 mm mm^{-1}) at a frequency of 0.5 Hz. No obvious mechanical fatigue was observed after being stretched for 1000 cycles. Scale bar = 5 mm.

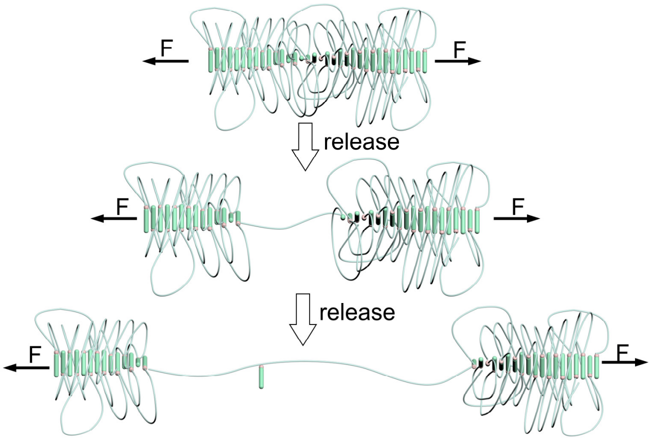

Supplementary Figure 11. Schematic of the step-by-step release of the hidden length upon rupture of neighbouring β strands in the picot fibres.

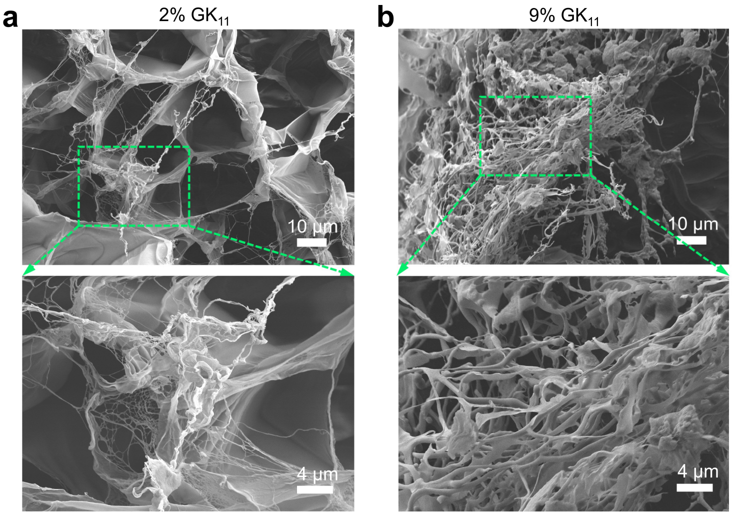

Supplementary Figure 12. FESEM images of p-Pep/Cu^{2+} hydrogels at different GK_{11} concentrations (2% and 9% w/v). a, FESEM images of p-Pep/Cu^{2+} hydrogels at the GK_{11} concentration of 2% w/v. b, FESEM images of p-Pep/Cu^{2+} hydrogels at the GK_{11} concentration of 9% w/v.

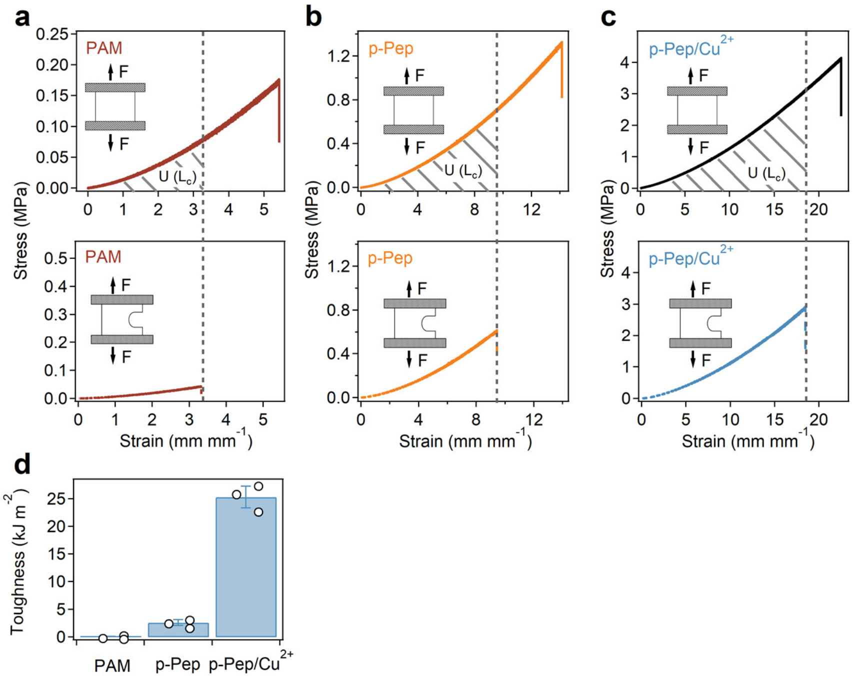

Supplementary Figure 13. Determination of the toughness (fracture energy) of different hydrogels. a-c, Stress–strain curves of notched and unnotched PAM (a), p-Pep (b) and p-Pep/Cu^{2+} (c) hydrogels. The insets illustrate the stretching of unnotched and notched samples. Shadings indicate the area below the tensile stress-strain curve of the

unnotched sample at the critical stretching distance L_c. d, Summary of toughness of PAM, p-Pep and p-Pep/Cu^{2+} hydrogels. The toughness of the PAM, p-Pep and p-Pep/Cu^{2+} hydrogels were 0.1 ± 0.01, 2.6 ± 0.6 and 25.3 ± 1.9 kJ m^{-2}, respectively. Values represent the mean and standard deviation (n = 3 independent samples).

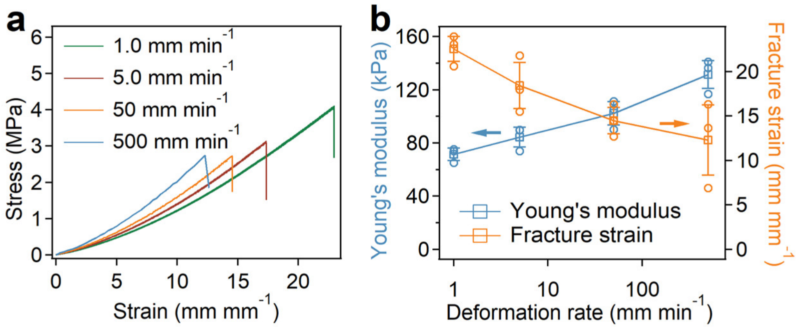

Supplementary Figure 14. Tensile mechanical properties of the p-Pep/Cu^{2+} hydrogels at different deformation rates. a, Uniaxial stress–strain curves of p-Pep/Cu^{2+} hydrogels under tension at tensile rates of 1.0, 5.0, 50, and 500 mm min^{-1}. b, Summaries of the Young's modulus and fracture strain of p-Pep/Cu^{2+} hydrogels at tensile rates of 1.0, 5.0, 50, and 500 mm min^{-1}. Values represent the mean and standard deviation (n = 3 independent samples).

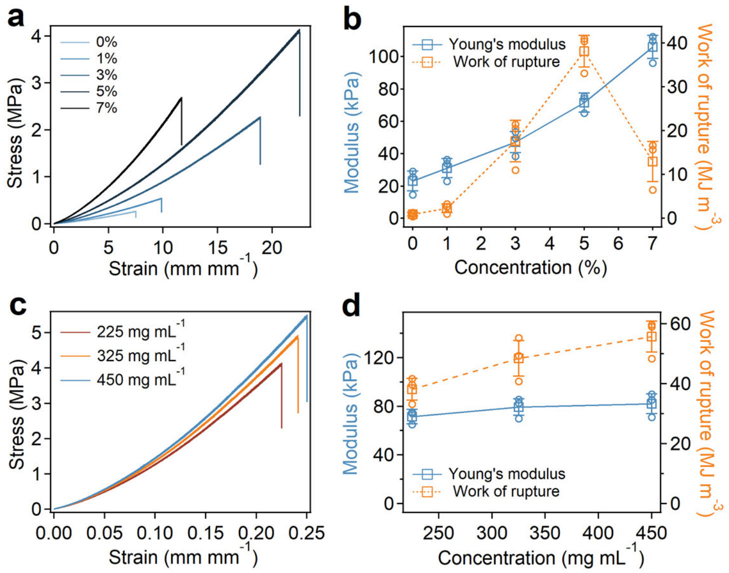

Supplementary Figure 15. Mechanical properties of the p-Pep/Cu^{2+} hydrogels with various 4-armed PEG-ACLT or acrylamide concentrations. a, Uniaxial stretching stress–strain curves of p-Pep/Cu^{2+} hydrogels with different 4-armed PEG-ACLT concentrations (0%, 1%, 3%, 5% and 7% w/v) in the precursors during preparation. b, Summaries of Young's modulus and work of rupture of p-Pep/Cu^{2+} hydrogels at different 4-armed PEG-ACLT concentrations (0%, 1%, 3%, 5% and 7% w/v) in the precursors during preparation. c, Uniaxial stretching stress–strain curves of p-Pep/Cu^{2+} hydrogels at different acrylamide concentrations in the precursors during preparation (225, 325 and 450 mg mL^{-1}). d, Summaries of Young's modulus and work of rupture of p-Pep/Cu^{2+} hydrogels at different acrylamide concentrations in the precursors during preparation (225, 325 and 450 mg mL^{-1}). For b and d, values represent the mean and standard deviation (n = 3 independent samples).

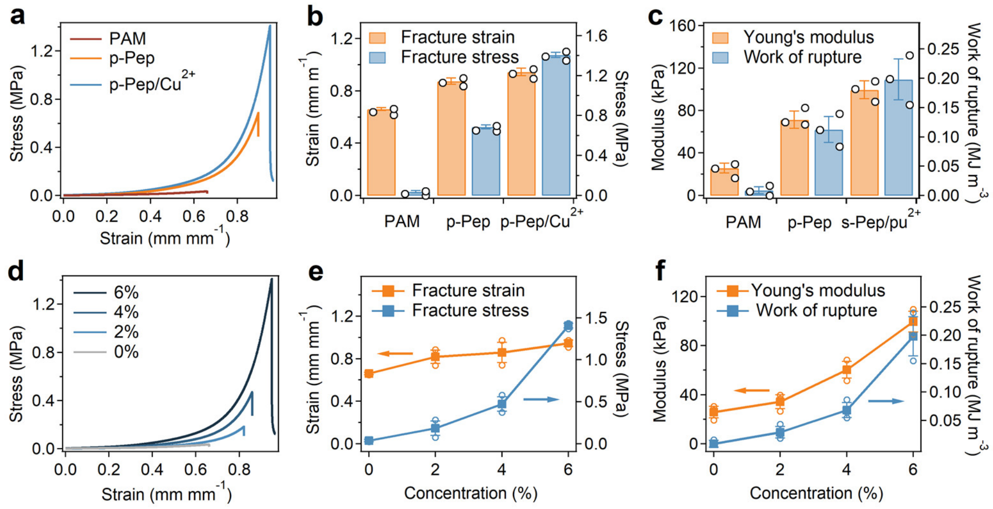

Supplementary Figure 16. Mechanical properties of p-Pep/Cu^{2+} hydrogels under compression. a, Typical stress–strain curves under compression for PAM, p-Pep and p-Pep/Cu^{2+} hydrogels. b, Summaries of fracture strain and stress of the PAM, p-Pep and p-Pep/Cu^{2+} hydrogels under compression. c, Summaries of Young's modulus and work of rupture of the PAM, p-Pep and p-Pep/Cu^{2+} hydrogels under compression. d, Typical stress–strain curves of p-Pep/Cu^{2+} hydrogels at various peptide concentrations in the precursor of hydrogels (0%, 2%, 4%, and 6% w/v) under compression. e, Summaries of fracture strain and stress of p-Pep/Cu^{2+} hydrogels at various peptide concentrations in the precursor of hydrogels (0%, 2%, 4%, and 6% w/v) under compression. f, Summaries of Young's modulus and work of rupture of p-Pep/Cu^{2+} hydrogels at various peptide concentrations in the precursor of hydrogels (0%, 2%, 4%, and 6% w/v) under compression. For b, c, e, and f, values represent the mean and standard deviation (n = 3 independent samples).

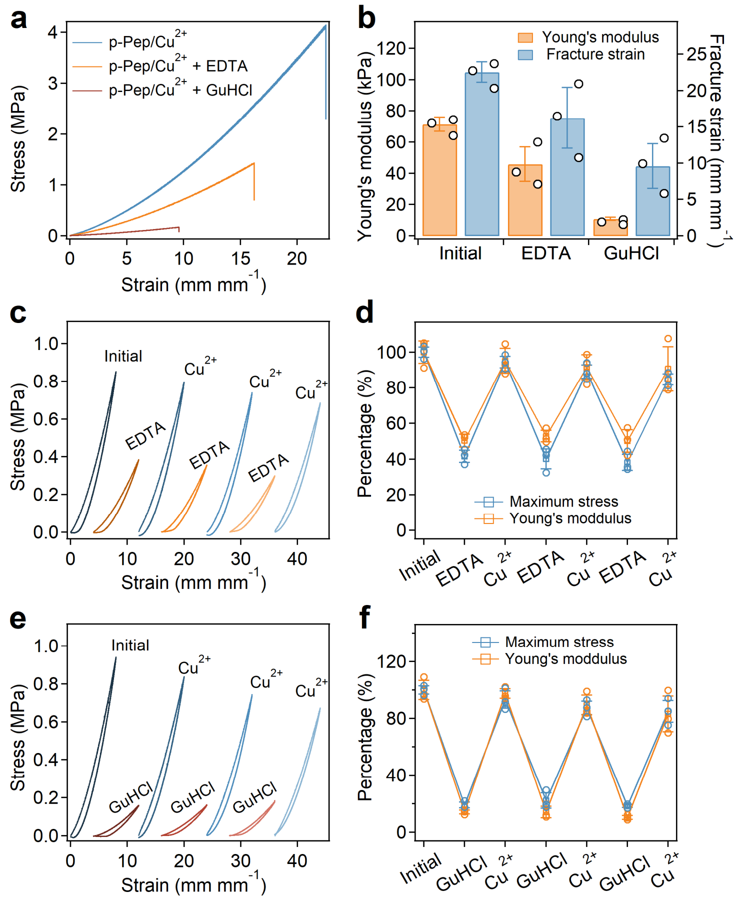

Supplementary Figure 17. Perturbing GK11-Cu^{2+} binding with EDTA and destroying peptide self-assembly with GuHCl. a, Tensile properties of the initial p-Pep/Cu^{2+} hydrogel, p-Pep/Cu^{2+} hydrogel treated with EDTA and p-Pep/Cu^{2+} hydrogel treated with GuHCl. b, Summaries of Young's moduli and fracture strains corresponding to the initial p-Pep/Cu^{2+} hydrogel, p-Pep/Cu^{2+} hydrogel treated with EDTA and p-Pep/Cu^{2+} hydrogel treated with GuHCl. c, Stretching-relaxation curves of p-Pep/Cu^{2+} hydrogels at a strain of 8 mm mm^{-1} in three cycles of perturbing the GK11-Cu^{2+} interaction with EDTA. d, Summaries of normalized Young's moduli and maximum stress of p-Pep/Cu^{2+} hydrogels in three cycles of perturbing the GK11-Cu^{2+} interaction with EDTA. e, Stretching-relaxation curves of p-Pep/Cu^{2+} hydrogels at a strain of 8 mm mm^{-1} in three cycles of destroying the peptide self-assembly with GuHCl. f, Summaries of normalized Young's moduli and maximum stress of p-Pep/Cu^{2+} hydrogels in three

cycles of destroying the peptide self-assembly with GuHCl. For b, d, and f, values represent the mean and standard deviation (n = 3 independent experiments).

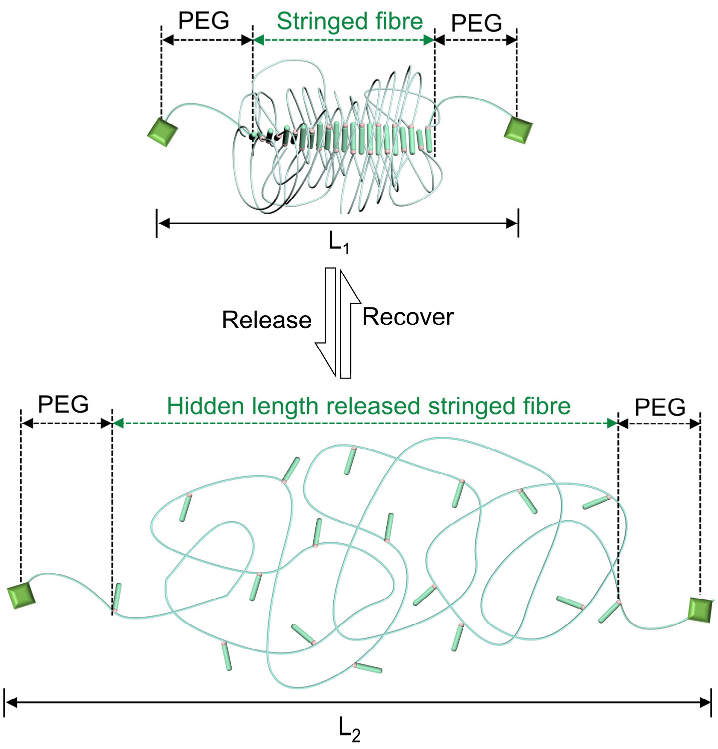

Supplementary Figure 18. Schematic of the release and recovery of the hidden length in picot fibres. According to the calculation based on the classical rubber elasticity theory and Gaussian chain statistics, the hidden length released between two neighbouring crosslinks (L_2) in the hydrogels was 1.8 times the initial length (L_1), which was close to the theoretical value of 2.0.

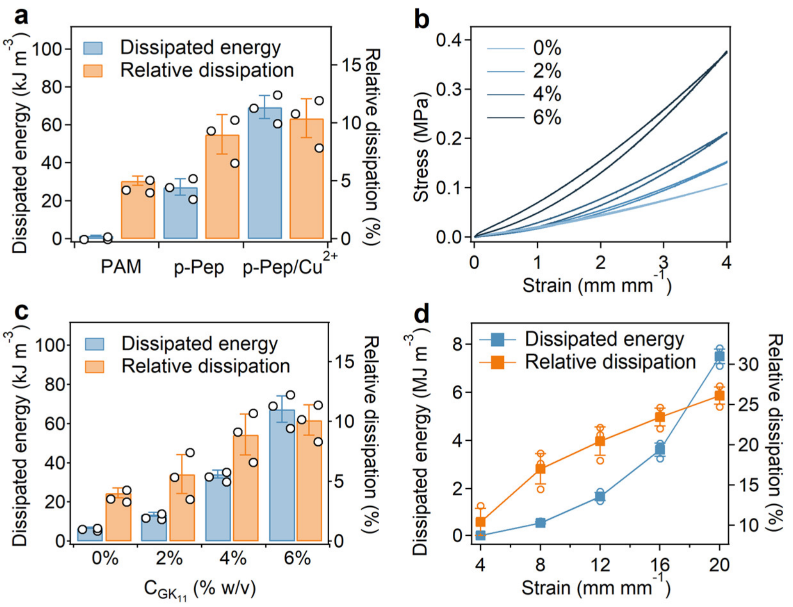

Supplementary Figure 19. Energy dissipation of PAM, p-Pep and p-Pep/Cu^{2+} hydrogels under tension. a, Dissipated energy and relative dissipation ratios of PAM, p-Pep and p-Pep/Cu^{2+} hydrogels at a strain of 4 mm mm^{-1}. b, Typical stretching-relaxation cycles of p-Pep/Cu^{2+} hydrogels (strain ~4 mm mm^{-1}) at various peptide concentrations in the precursor of hydrogels (0%, 2%, 4%, and 6% w/v). c, Summaries of dissipated energy and relative dissipation ratios of p-Pep/Cu^{2+} hydrogels (strain ~4 mm mm^{-1}) at various peptide concentrations in the precursor of hydrogels (0%, 2%, 4%, and 6% w/v). d, Summaries of dissipated energy and relative dissipation ratios of p-Pep/Cu^{2+} hydrogels at various strains (4-20 mm mm^{-1}). For a, c, and d, values represent the mean and standard deviation (n = 3 independent samples).

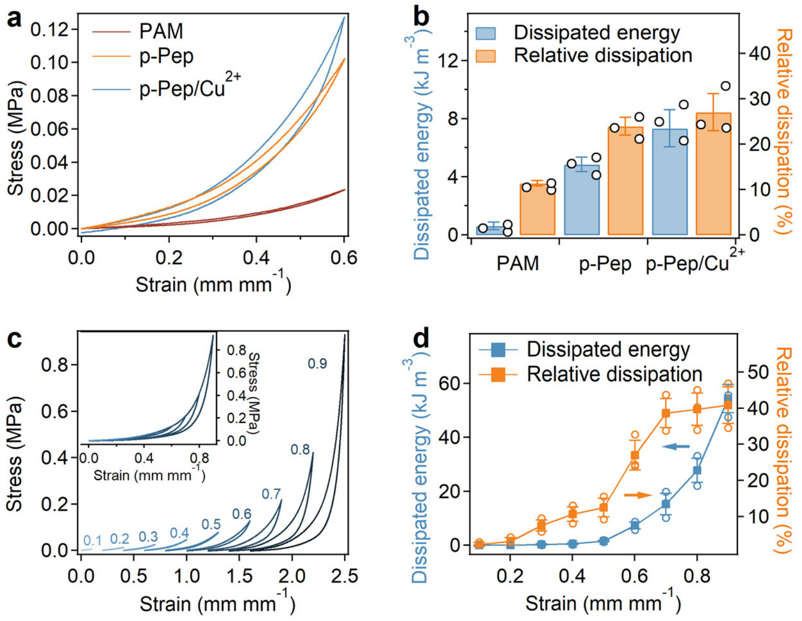

Supplementary Figure 20. Energy dissipation of PAM, p-Pep and p-Pep/Cu^{2+} hydrogels under compression. a, Typical compression-relaxation cycles of PAM, p-Pep and p-Pep/Cu^{2+} hydrogels at a strain of 0.6 mm mm^{-1}. b, Summaries of dissipated energy and relative dissipation ratios of PAM, p-Pep and p-Pep/Cu^{2+} hydrogels at a strain of 0.6 mm mm^{-1}. c, Compressing-relaxation cycles of p-Pep/Cu^{2+} hydrogels at various strains (0.1-0.9 mm mm^{-1}). The curves have been offset for clarity, and the overlapping curves are shown in the inset. d, Summaries of dissipated energy and relative dissipation ratios of p-Pep/Cu^{2+} hydrogels at various strains (0.1-0.9 mm mm^{-1}) under compression. For b and d, values represent the mean and standard deviation (n = 3 independent samples).

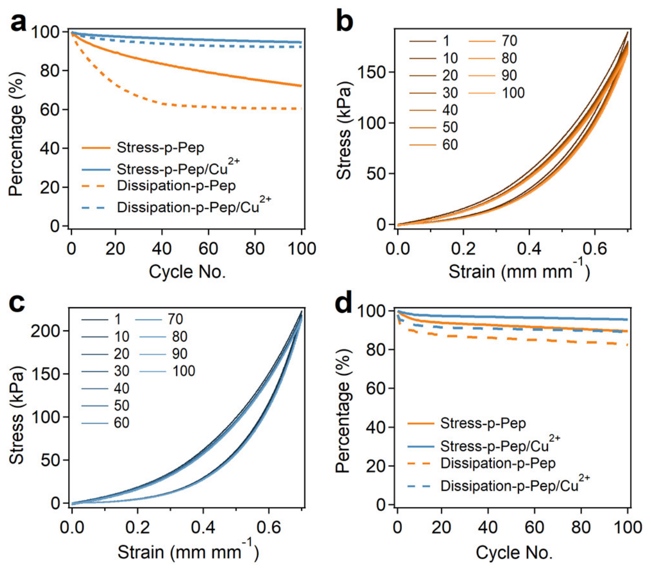

Supplementary Figure 21. Recovery of hydrogels during consecutive deformation-relaxation cycles. a, Normalized maximum stress and energy dissipation of p-Pep and p-Pep/Cu^{2+} hydrogels corresponding to consecutive stretching-relaxation cycles as in Fig. 4e. b, c, Stress–strain curves of 100 consecutive compression-relaxation cycles without any waiting time for p-Pep (b) and p-Pep/Cu^{2+} (c) hydrogels at a strain of 0.7 mm mm^{-1}. d, Normalized maximum stress and energy dissipation of p-Pep and p-Pep/Cu^{2+} hydrogels corresponding to consecutive compression-relaxation cycles in b and c.

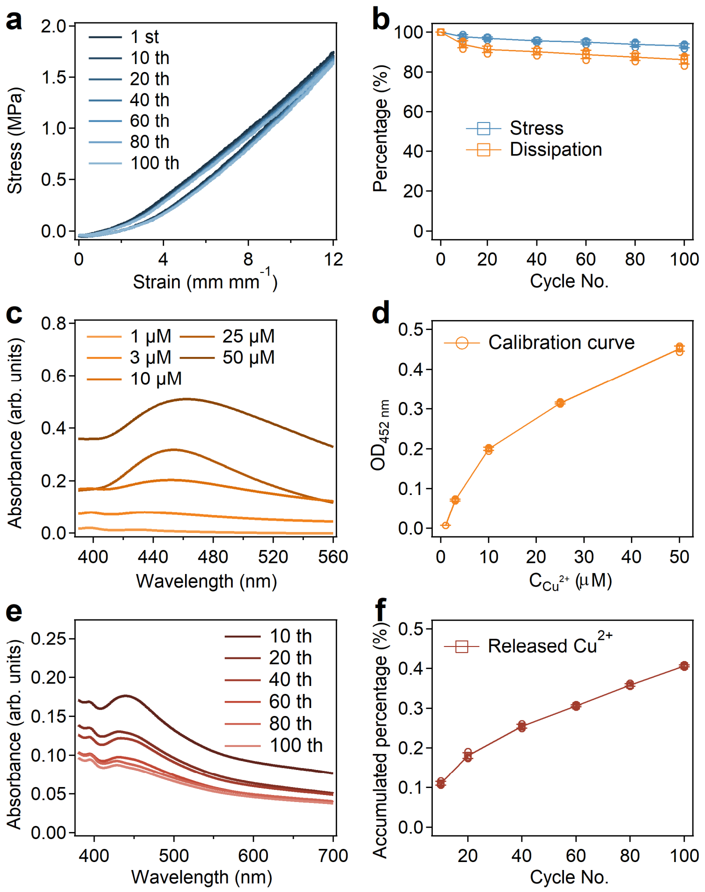

Supplementary Figure 22. Mechanical property and Cu^{2+} release of p-Pep/Cu^{2+} hydrogels under stretching-relaxation cycles in Tris buffer (pH=7.60, containing 300 mM KCl). a, Typical stress–strain curves for p-Pep and p-Pep/Cu^{2+} hydrogels under stretching-relaxation cycles. The cycle numbers are 1, 10, 20, 40, 60, 80 and 100. b, Summary of the maximum stress and energy dissipation for p-Pep/Cu^{2+} hydrogels under stretching-relaxation cycles. c, UV-vis spectra for mixture of sodium diethyldithiocarbamatre (DDTC-Na) and Cu^{2+} at different concentrations of Cu^{2+} (1-50 μM). The concentration of DDTC-Na was 250 μM. d, Calibration curve of Cu^{2+} concentrations and OD_{452nm} (UV-vis absorbance at 452 nm). e, UV-vis spectra of the hydrogel leachates after different cycles in the presence of DDTC-Na. f, Accumulated percentage of released Cu^{2+} from the p-Pep/Cu^{2+} hydrogels after different cycles. For b, d and f, values represent the mean and standard deviation (n = 3 independent experiments).

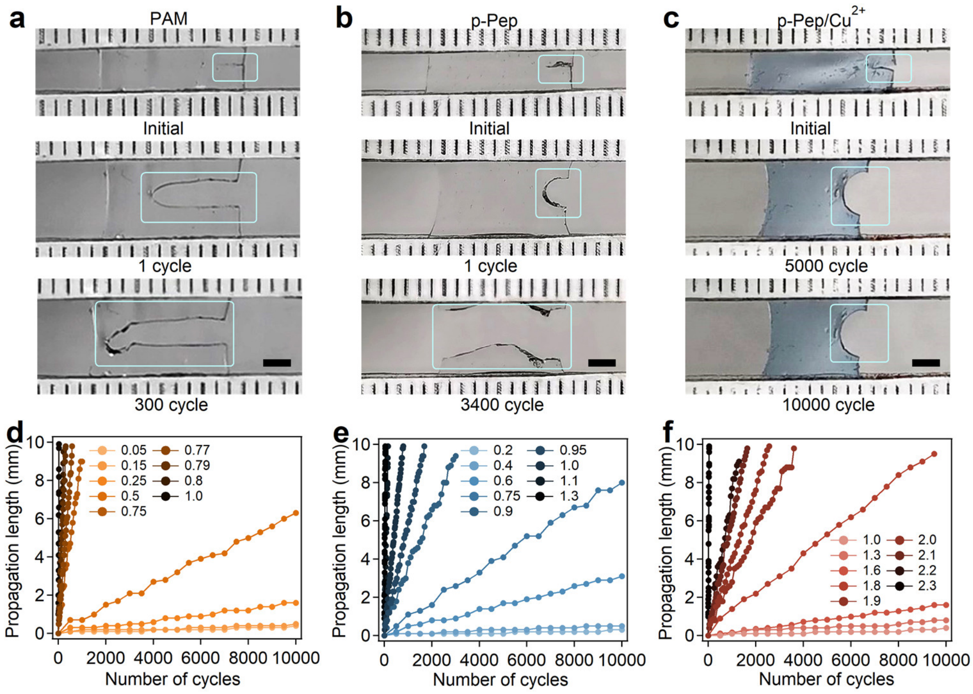

Supplementary Figure 23. Fatigue resistance of three different hydrogels. a-c, Images of PAM (a), p-Pep (b) and p-Pep/Cu^{2+} hydrogels (c) after different numbers of stretch-relaxation cycles at a strain of 1.0 mm mm^{-1}. Scale bar = 2 mm. d-f, Crack propagation after different cycles of stretching-relaxation at different applied strains for the PAM (d), p-Pep (e) and p-Pep/Cu^{2+} (f) hydrogels.

Supplementary Tables

Supplementary Table 1. Tensile mechanical properties of PAM, p-Pep and p-Pep/Cu^{2+} hydrogels containing different concentrations of peptides. Values represent the mean and standard deviation.

	Peptide concentration (v/w)	Fracture strain (mm mm^{-1})	Fracture stress (MPa)	Young's modulus (kPa)	Work of rupture (MJ m^{-3})	Toughness (kJ m^{-2})
PAM	/	5.4 ± 0.8	0.18 ± 0.03	13.2 ± 3.2	0.4 ± 0.04	0.10 ± 0.01
p-Pep	6%	14.1 ± 0.9	1.35 ± 0.12	25.4 ± 5.9	7.3 ± 1.6	2.6 ± 0.6
p-Pep/Cu^{2+}	2%	7.6 ± 0.3	0.40 ± 0.03	20.9 ± 3.7	1.3 ± 0.4	--
	4%	16.8 ± 0.7	1.92 ± 0.17	29.5 ± 2.8	12.6 ± 1.6	--
	6%	22.5 ± 1.4	4.12 ± 0.37	71.5 ± 4.6	38.1 ± 3.6	25.3 ± 1.9
	9%	18.2 ± 1.2	3.66 ± 0.21	121.5± 14.0	29.1 ± 3.5	--

Supplementary Table 2. Mechanical properties of the p-Pep/Cu^{2+} hydrogel, and other tough and rapid recovery hydrogels from the literature.

Classification	Sample code	Water content (wt%)	Young's modulus (MPa)	Fracture strain (mm/mm)	Fracture stress (MPa)	Work of rupture (MJ m^{-3})	Toughness (kJ m^{-2})	Recovery time (min)	Recovery efficiency (%)	Ref. No.
	p-Pep/Cu^{2+}	87<br>82 (*)	0.07<br>0.1 (*)	22.5<br>25.0 (*)	4.12<br>5.47 (*)	38.1<br>55.7 (*)	25.3<br>--	0<br>--	~100<br>--	This work
Single-network hydrogel	Highly entangled hydrogel	~70	0.05	~4.5	0.4	--	2.2	--	--	23
	Polyampholytes gel	50-70	0.01-8	1.5-15	0.1-2	0.1-7.0	1.0-4.0	120	~100	24
	$(FL)_8$ gel	70	0.016	4.5	0.035	--	--	20	~85	25
	DMAA-MAAc hydrogel	28	8	8	2	--	9.3	3 (37 °C)	~100	26
	P(urea-IL_a-$SPMA_b$)-3d gel	~50	1.97	4.78	1.90	6.70	--	120	~85	27
	PIC gel	55	5.4	7.5	3.8	18.8	10.0	120	85	28
Dual-crosslinked hydrogel	CB[8] gel	90	0.0046	24	~130	--	0.75	3	~100	29
	$HN-PH_6$	80.4	0.27	4.12	3.02	4.03	--	0	~85	30
	D-hydrogel-0.15	60-70	~1.75	7.48	5.9	27.2	--	240	87.6	31
	CCP-MCP1 gel	32	0.145	5.49	2.6	--	1.33	>15	--	32
Double-network hydrogel	Ca^{2+}-alginate-PAAm	86	0.029	23	0.156	--	8.7	1440	74	14
	B-DN3 gel	44	22	5.7	10.5	--	2.85	5	~85	33
	Agar/PAMAAc-Fe^{3+} DN	--	0.27	14	1.55	16.7	0.894	20	95	34
	DN-Sul gel	54.6	0.8	5.05	3.7	7.6	9.8	240	>90	35
	DN-Cit gel	56.9	1.3	5	5.6	12.1	14	240	96.6	35
	PAM-CS-S DN gel	~80	0.357	5.6	1.94	--	8.3	240	90	36
Nanocomposite hydrogel	L-NC gel	62	43.2	7.4	1.6	7.38	--	--	--	37
	SHARK hydrogel		0.03	77	1.02	32.6	19.75	--	--	38

(*) The concentration of acrylamide in the precursor of hydrogels was 450 mg mL^{-1}.

Supplementary References:

1 Jumper, J. *et al.* Highly accurate protein structure prediction with AlphaFold. *Nature* **596**, 583-589 (2021).

2 Mirdita, M. *et al.* ColabFold: making protein folding accessible to all. *Nat. Methods* **19**, 679-682 (2022).

3 Fidler, D. R. *et al.* Using HHsearch to tackle proteins of unknown function: A pilot study with PH domains. *Traffic* **17**, 1214-1226 (2016).

4 Bhadra, P. & Siu, S. W. I. Refined Empirical Force Field to Model Protein-Self-Assembled Monolayer Interactions Based on AMBER14 and GAFF. *Langmuir* **35**, 9622-9633 (2019).

5 Hess, B., Kutzner, C., van der Spoel, D. & Lindahl, E. GROMACS 4: Algorithms for Highly Efficient, Load-Balanced, and Scalable Molecular Simulation. *J. Chem. Theory Comput.* **4**, 435-447 (2008).

6 Miyamoto, S. & Kollman, P. A. Settle: An analytical version of the SHAKE and RATTLE algorithm for rigid water models. *J. Comput. Chem.* **13**, 952-962 (1992).

7 Hess, B., Bekker, H., Berendsen, H. J. C. & Fraaije, J. G. E. M. LINCS: A linear constraint solver for molecular simulations. *J. Comput. Chem.* **18**, 1463-1472 (1997).

8 Lin, Y.-F. *et al.* MIB: Metal Ion-Binding Site Prediction and Docking Server. *J. Chem. Inf. Model.* **56**, 2287-2291 (2016).

9 Miller, B. R., 3rd *et al.* MMPBSA.py: An Efficient Program for End-State Free Energy Calculations. *J. Chem. Theory Comput.* **8**, 3314-3321 (2012).

10 Case, D. *et al. Amber 2015, University of California, San Francisco*. (2015).

11 Sun, T. L. *et al.* Physical hydrogels composed of polyampholytes demonstrate high toughness and viscoelasticity. *Nat. Mater.* **12**, 932-937 (2013).

12 Tanaka, Y. *et al.* Determination of fracture energy of high strength double network hydrogels. *J. Phys. Chem. B* **109**, 11559-11562 (2005).

13 Jackson, A. P. Measurement of the fracture toughness of some contact lens hydrogels. *Biomaterials* **11**, 403-407 (1990).

14 Sun, J.-Y. *et al.* Highly stretchable and tough hydrogels. *Nature* **489**, 133-136 (2012).

15 Oesterhelt, F., Rief, M. & Gaub, H. E. Single molecule force spectroscopy by AFM indicates helical structure of poly(ethylene-glycol) in water. *New J. Phys.* **1**, 6-6 (1999).

16 Li, B. *et al.* Single-Molecule Force Spectroscopy Reveals Self-Assembly Enhanced Surface Binding of Hydrophobins. *Chem-Eur. J.* **24**, 9224-9228 (2018).

17 Lee, H., Venable, R. M., MacKerell, A. D. & Pastor, R. W. Molecular Dynamics Studies of Polyethylene Oxide and Polyethylene Glycol: Hydrodynamic Radius and Shape Anisotropy. *Biophys. J.* **95**, 1590-1599 (2008).

18 Giannotti, M. I. & Vancso, G. J. Interrogation of single synthetic polymer chains and polysaccharides by AFM-based force spectroscopy. *Chemphyschem* **8**, 2290-2307 (2007).

19 Fu, L. & Li, H. Toward Quantitative Prediction of the Mechanical Properties of Tandem Modular Elastomeric Protein-Based Hydrogels. *Macromolecules* **53**, 4704-4710 (2020).

20 James, H. M. & Guth, E. Theory of the Elastic Properties of Rubber. *J. Chem. Phys.* **11**, 455-481 (1943).

21 Anseth, K. S., Bowman, C. N. & Brannon-Peppas, L. Mechanical properties of hydrogels and their experimental determination. *Biomaterials* **17**, 1647-1657 (1996).

22 Gosline, J. M., Denny, M. W. & DeMont, M. E. Spider silk as rubber. *Nature* **309**, 551-552 (1984).

23 Kim, J., Zhang, G., Shi, M. & Suo, Z. Fracture, fatigue, and friction of polymers in which entanglements greatly outnumber cross-links. *Science* **374**, 212-216 (2021).

24 Sun, T. L. *et al.* Physical hydrogels composed of polyampholytes demonstrate high toughness and viscoelasticity. *Nat. Mater.* **12**, 932-937 (2013).

25 Fang, J. *et al.* Forced protein unfolding leads to highly elastic and tough protein hydrogels. *Nat. Commun.* **4**, 2974 (2013).

26 Hu, X., Vatankhah-Varnoosfaderani, M., Zhou, J., Li, Q. & Sheiko, S. S. Weak Hydrogen Bonding Enables Hard, Strong, Tough, and Elastic Hydrogels. *Adv. Mater.* **27**, 6899-6905 (2015).

27 Long, T., Li, Y., Fang, X. & Sun, J. Salt-Mediated Polyampholyte Hydrogels with High Mechanical Strength, Excellent Self-Healing Property, and Satisfactory Electrical Conductivity. *Adv. Funct. Mater.* **28**, 1804416 (2018).

28 Luo, F. *et al.* Oppositely Charged Polyelectrolytes Form Tough, Self-Healing, and Rebuildable Hydrogels. *Adv. Mater.* **27**, 2722-2727 (2015).

29 Liu, J. *et al.* Biomimetic Supramolecular Polymer Networks Exhibiting both Toughness and Self-Recovery. *Adv. Mater.* **29**, 1604951 (2017).

30 Sun, W. *et al.* Molecular engineering of metal coordination interactions for strong, tough, and fast-recovery hydrogels. *Sci. Adv.* **6**, eaaz9531 (2020).

31 Lin, P., Ma, S., Wang, X. & Zhou, F. Molecularly Engineered Dual-Crosslinked Hydrogel with Ultrahigh Mechanical Strength, Toughness, and Good Self-Recovery. *Adv. Mater.* **27**, 2054-2059 (2015).

32 Gonzalez, M. A. *et al.* Strong, Tough, Stretchable, and Self-Adhesive Hydrogels from Intrinsically Unstructured Proteins. *Adv. Mater.* **29**, 1604743 (2017).

33 Zhang, H. J. *et al.* Tough Physical Double-Network Hydrogels Based on Amphiphilic Triblock Copolymers. *Adv. Mater.* **28**, 4884-4890 (2016).

34 Chen, Q. *et al.* Improvement of Mechanical Strength and Fatigue Resistance of Double Network Hydrogels by Ionic Coordination Interactions. *Chem. Mater.* **28**, 5710-5720 (2016).

35 Yang, Y., Wang, X., Yang, F., Wang, L. & Wu, D. Highly Elastic and Ultratough Hybrid Ionic–Covalent Hydrogels with Tunable Structures and Mechanics. *Adv. Mater.* **30**, 1707071 (2018).

36 Yang, Y., Wang, X., Yang, F., Shen, H. & Wu, D. A Universal Soaking Strategy to Convert Composite Hydrogels into Extremely Tough and Rapidly Recoverable Double-Network Hydrogels. *Adv. Mater.* **28**, 7178-7184 (2016).

37 Wang, J., Lin, L., Cheng, Q. & Jiang, L. A Strong Bio-Inspired Layered PNIPAM–Clay Nanocomposite Hydrogel. *Angew. Chem. Int. Edit.* **51**, 4676-4680 (2012).

38 Xue, B. *et al.* Stretchable and self-healable hydrogel artificial skin. *Natl. Sci. Rev.* **9** (2021).
