# Supplementary material for: Strong, tough, rapid-recovery, and fatigue-resistant hydrogels made of picot peptide fibres
Source: Nat Commun. 2023 May 4;14:2583. doi: 10.1038/s41467-023-38280-4 (PMC10160100; doi:10.1038/s41467-023-38280-4)
Supplement: Supplementary file 3 — Description of Additional Supplementary Files [file 41467_2023_38280_MOESM3_ESM.pdf]

Description of Additional Supplementary Files

File Name: Supplementary Movie 1
Description: Rapid recovery of p-Pep/Cu^{2+} hydrogel at different compression strains.

File Name: Supplementary Movie 2
Description: Compressing p-Pep/Cu^{2+} hydrogel with a sharp blade.

File Name: Supplementary Movie 3
Description: Continuously stretching the p-Pep/Cu^{2+} hydrogel 1000 times at a frequency of 1 Hz.
